# Chronic Cough as a Genetic Neurological Disorder? Insights from Cerebellar Ataxia with Neuropathy and Vestibular Areflexia Syndrome (CANVAS)

**DOI:** 10.1007/s00408-023-00660-4

**Published:** 2023-11-18

**Authors:** Richard D. Turner, Barnaby Hirons, Andrea Cortese, Surinder S. Birring

**Affiliations:** 1 Department of Respiratory Medicine, Gold Coast University Hospital, Southport, QLD Australia; 2 School of Medicine and Dentistry, Griffith University, Southport, QLD Australia; 3 Department of Respiratory Medicine, King’s College Hospital, London, UK; 4 Centre for Human and Applied Physiological Sciences, School of Basic and Medical Biosciences, King’s College London, London, UK; 5 Department of Neuromuscular Disease, UCL Queen Square Institute of Neurology, London, UK; 6 Department of Brain and Behaviour Sciences, University of Pavia, Pavia, Italy

**Keywords:** CANVAS, RFC1, Chronic cough

## Abstract

Chronic cough is common, and in many cases unexplained or refractory to otherwise effective treatment of associated medical conditions. Cough hypersensitivity has developed as a paradigm that helps to explain clinical and research observations that frequently point towards chronic cough as a neuropathic disorder. Cerebellar ataxia with neuropathy and vestibular areflexia syndrome (CANVAS) is a recently described neurological condition whose clinical features include gait ataxia, unsteadiness, peripheral neuropathy, and autonomic dysfunction. Chronic cough is also a common feature of the syndrome, with features of hypersensitivity, often preceding core neurological symptoms by up to 30 years or more. The genetic basis in a majority of cases of CANVAS appears to be biallelic variable repeat intron expansion sequences within *RFC1*, a gene normally involved in the regulation of DNA replication and repair. The same polymorphism has now been identified at an increased frequency in patients with unexplained or refractory chronic cough in the absence of defining clinical features of CANVAS. This review expands on these points, aiming to increase the awareness of CANVAS amongst clinicians and researchers working with chronic cough. We discuss the implications of a link between *RFC1* disease and cough. Improved understanding of CANVAS may lead to an enhanced grasp of the pathophysiology of chronic cough, and new approaches to antitussive treatments.

## Introduction

Chronic cough is an irksome condition, primarily for the sufferer and those around them, but also for the treating health professional for whom there are often few effective therapeutic options. It may be present in approximately 10% of general populations and has significant impact on quality of life, partly from secondary physical effects such as rib pain and urine incontinence, and psychological and social effects limiting interactions with others [1, 2]. There have been significant advances in understanding of chronic cough over the last decades, leading to detailed evidence-based guidelines [3]. However, once coughs exacerbated by more tractable factors such as undertreated asthma or angiotensin-converting enzyme inhibitor medications have been addressed, many patients remain inadequately treated. Up to > 40% of patients attending specialist clinics have either unexplained or refractory chronic cough (UCC or RCC) [4]. Such coughs persist, often over many years or decades, in either the absence of detectable contributory pathology (for UCC), or, in the case of RCC, the otherwise effective treatment of coexisting aggravating conditions such as asthma, gastro-oesophageal reflux disease and chronic sinusitis [3]. Recently, the paradigm of chronic cough has begun to shift from being considered merely a manifestation of other disease, to the idea that, in many cases, the cough itself may be the disease, a direct consequence of pathological dysfunction of the normal physiological processes which control cough in healthy individuals [5].

Cerebellar ataxia with neuropathy and vestibular areflexia syndrome, or CANVAS, is a progressive late-onset chronic neurological disorder only fully described within the last 12 years [6, 7]. Amongst its associated characteristic clinical features is chronic cough, often preceding other symptoms by many years. In 2019 biallelic AAGGG repeat expansions in the gene *RFC1* gene were identified as the cause of CANVAS, and a common cause of late onset ataxia and sensory neuropathy with cough [8, 9]. Subsequently, *RFC1* expansions were identified in an unusually high proportion of patients with UCC/RCC in the absence of the classical neurological features of the syndrome [10]. What follows is a review of the current thinking of the mechanisms underlying chronic cough, and how this might fit with the existence of a neurological disease, apparently genetically driven, which has cough as a frequent feature.

## Existing Concept of Refractory + Unexplained Chronic Cough

Common to all suffers of unexplained or refractory chronic cough is repeated episodes of coughing, often in the presence of either minimal, or no discernible triggers. Terms have been coined to describe these and related phenomena; hypertussia for an exaggerated cough reflex, and allotussia for coughing with a stimulus that in healthy individuals would be insufficient to produce a response [5]. Associated with this is often a persistent sensation of ‘tickle in the throat’, or overwhelming urge to cough, described as laryngeal paraesthesia [11]. Objectively, responses to tussigenic compounds such as capsaicin or citric acid in inhalation challenge tests are increased in individuals with UCC/RCC compared to healthy controls, with a reduction in the threshold concentration of compound required to elicit a given number of coughs [12].

These clinical and experimental observations have led to the increasingly popular concept of cough hypersensitivity as a syndrome associated with (or underlying) much of unexplained and refractory chronic cough [13]. Implicit in this paradigm is the idea of chronic cough as neuropathic disorder [5]. This in turn is supported by a possible increased prevalence of autonomic dysfunction in association with chronic cough [14], and the proven efficacy of neuromodulator medications in treating the condition. Such drugs, including gabapentin [15], pregabalin [16], and amitriptyline [17], are thought to exert their effects through synaptic neurotransmission, and are well-established treatments for neuropathic pain [18].

Much of the focus on mechanism for UCC/RCC has been on the afferent arc of the cough reflex. The condition has been shown to be associated with changes in the characteristics of airway sensory nerves, such as their density [19], and the expression of cell membrane channel receptors including TRPV1 [20]. However, centrally located neural pathways are likely to also play a key part. Supportive evidence for this includes the observation that individuals with UCC appear to have a reduced ability to voluntarily suppress cough responses to inhaled capsaicin compared to healthy controls [21]. In turn, functional brain imaging in patients with clinical cough hypersensitivity, has demonstrated reduced levels of activity in specific cortical areas during attempts at voluntarily suppressing cough [22].

The concept of cough hypersensitivity due to underlying neuropathy is attractive as it fits with clinical observations, but significant gaps in knowledge as to responsible mechanisms remain. The failure of a TRPV1 receptor agonist to reduce cough frequency in patients with chronic cough despite blunting the tussive response to inhaled capsaicin also illustrates the complexity of chronic cough, potentially with a redundancy of mechanistic pathways [23].

## Cerebellar Ataxia with Neuropathy and Vestibular Areflexia Syndrome

CANVAS was first so-named in 2011 to refer to a series of patients with late-onset slowly progressing cerebellar ataxia, bilateral vestibular areflexia, and axonal sensory neuropathy [24]. Orthostatic hypotension was also noted to be a common feature in many such individuals [25], as was neuropathic pain and chronic cough [26].

### Clinical Features

The clinical features of CANVAS are summarised in the Figure. The median age of onset of core neurological symptoms is 52 years, and males and females appear to be affected equally [27]*.* The presence of pairs of sibling cases in original case series was an early pointer to a genetic basis of possible recessive inheritance, as discussed further below [28]. Initial neurological symptoms are sensory loss or paraesthesia, unsteadiness, poor balance and falls (often more prominent in the dark), dizziness and oscillopsia [29]. Dysarthria and dysphasia can then develop, usually as later features, along with orthostatic hypotension and other autonomic symptoms such as dry mouth and eyes, urinary retention, and erectile dysfunction [30]. As below, chronic cough associated with CANVAS can pre-date classical neurological symptoms by 30 years [27, 31]. Neuropathic pain is also common, and can be particularly troublesome, with allodynia and dysaethesia [26, 32].

Ataxia is due to a combination of cerebellar dysfunction and an axonal neuropathy-driven defective proprioception, with or without a failure of vestibular function. The relative timing of onset of each of the three components is variable, contributing to delay in diagnosis of CANVAS [6].

Clinical signs include those of a cerebellar disorder such as gaze-evoked nystagmus, broken pursuits, dysarthria, and dysphagia (Table 1). Bilateral vestibular impairment can be demonstrated by a reduced or absent vestibular-ocular reflex, such as through a failure of eye stabilisation during head rotation, tested clinically by the head impulse test [6, 33]. Proprioception defects manifest clinically as abnormal vibration and joint position sense testing.

**Table 1:** Clinical assessment and management of CANVAS

Symptom	Examination	Investigation	Differential diagnosis	Treatment in CANVAS
Unsteadiness: - cerebellar - vestibular - somatosensory	Ataxia: - broad-based gait - nystagmus - impaired coordination - dysarthria - dysphagia VOR impairment: - bilateral catch-up saccades on HIT Romberg’s + ve	MRI brain: - Cerebellar atrophy Videofluoroscopy vHIT/VNG - bilateral vestibular hypofunction MRI spine - cord atrophy - T2 hyperintensity in posterior column	Ataxia: - genetic, CVA, MS, trauma, alcohol, vitamin deficiency, SOL, toxins, medications VOR impairment: - Meniere’s disease - ototoxicity - post-meningitis - Idiopathic	Physiotherapy Speech therapy Occupational therapy
Peripheral neuropathy: - sensory loss - paraesthesia - pain	Impaired pinprick sense, vibration sense, proprioception	NCS: - absent / reduced sensory potentials - normal motor studies	Multiple: idiopathic, metabolic, drugs, genetic, paraneoplastic, infection, vascular, vitamin deficiency	Neuromodulator medications* for neuropathic pain Physiotherapy
Oscillopsia	HIT Nystagmus	vHIT / VNG	VOR impairment Acquired nystagmus Brainstem-cerebellar disease	Vestibular rehabilitation Head-eye coordination exercises
Dysautonomia	Lying/standing BP and HR Cardiac: arrhythmias, tachycardia Dermatology: vasomotor changes, Raynaud’s, anhidrosis	Tilt table test Valsalva manoeuvre HRV to paced breathing Urodynamics Gastric emptying studies	Multiple: primary or secondary; diabetes, MS, POTS, paraneoplastic, alcohol, autoimmune, Parkinson’s, MSA	As per symptom: lifestyle, medication, external compression

*MRI* magnetic resonance imaging, *HIT* head impulse test, *vHIT* video HIT, *VNG* videonystagmography, *CVA* cerebrovascular accident, *MS* multiple sclerosis, *POTS* postural orthostatic tachycardia syndrome, *MSA* multiple system atrophy, *SOL* space occupying lesion, *VOR* vestibulo-ocular reflex, *NCS* nerve conduction studies, *HR* heart rate, *HRV* heart rate variability

*Pregabalin, gabapentin, duloxetine, amitriptyline. As referenced from Refs [27, 29–31]

The neurological deficits of CANVAS usually progress slowly but irreversibly over years, and clinical management is currently supportive. In one case series the onset of autonomic dysfunction was apparent a median of five years after the appearance of gait ataxia, dysarthria at 8 years, and dysphagia at 10 years, with substantial variability in the timing and the severity of each, including in the need for walking aids or wheelchair dependence [34].

### Neurophysiology and -Pathology

Peripheral sensory nerve conduction is invariably abnormal in established cases of CANVAS, and to a greater degree in the lower limbs than upper, whereas motor conduction is largely unimpaired [27]. Spinal cord and cerebellar atrophy is commonly seen on magnetic resonance imaging.

Muscle histology is typically normal, whilst sensory nerve biopsy demonstrates a substantial reduction in numbers of large and small myelinated fibres, an absence of axonal regeneration, and no evidence of demyelination [27]. Descriptions of postmortem examinations have noted changes focussed on the cerebellum and spinal cord. In particular, there is substantial dorsal root ganglionopathy and depletion of cerebellar Purkinje cells, especially obvious in the vermis [35]. In keeping with autonomic dysfunction there was also loss of neurones within the vagal nucleus in one report [36].

### Genetics

Independent work by two separate groups, through linkage analysis, whole genome sequencing and other techniques, has recently identified specific polymorphisms in the gene encoding replication factor complex subunit 1 (*RFC1*) in > 90% of both sporadic and familial cases of clinically diagnosed CANVAS [8, 9]. Cases possessed biallelic (homozygous) AAGGG repeat expansion sequences within the second intron of *RFC1* [37]. This finding has subsequently been replicated in other case series of varied ethnicity, although with some variations in the specific expanded pentanucleotide repeat sequence including uninterrupted ACAGG, AAAGG and AGGGC and mixed motif AAAGG-AAGGG and AGAGG-AAAGG [7, 38–40].

The normal function of *RFC1* relates to coordinating DNA repair and replication [41]. In particular, the five-subunit gene product replication factor C is termed a clamp loader, functioning to load ring-shaped sliding clamps onto replicating DNA, which in turn tether polymerases to facilitate nucleic acid chain extension [42]. However, it is as yet unknown exactly how specific *RFC1* variants lead to disease. The presence of the repeat expansion sequence is hypothesised to alter the three-dimensional DNA structure of *RFC1*. Although > 100 repeats of the abnormal motif appear to be required to cause disease, it seems to be the nature of the particular repeated pentanucleotide motif rather than the absolute number of repeats that is responsible for pathology [37]. Despite this, no alteration in the *RFC1* RNA transcript or protein product has yet been identified in individuals with clinically defined CANVAS, and understanding of the process of disease development remains poor [8]. However, it is of interest that mutations in other genes of DNA repair are associated with several neurodegenerative disorders including xeroderma pigmentosum and ataxia-telangiectasia [43]. Ataxia and neuropathy are common to all of these, suggesting a particular vulnerability of peripheral nerves and the cerebellum to DNA damage.

Following the identification of the putative genetic basis of CANVAS, a wider category of *RFC1*-associated late-onset neurological syndromes has been identified [37]. The majority lie along a spectrum which includes CANVAS, including isolated axonal sensory neuropathy [44]*,* and complex cerebellar ataxia with neuropathy [45]. A few cases who developed Parkinsonism or cognitive dysfunction in later disease stages were have also been reported [37]. The penetrance and extent of variable expressivity of pathogenic biallelic AAGGG repeat expansion sequences is not clear. In European populations the allele frequency of *RFC1* AAGGG expansion is estimated at ~ 1–5% of healthy individuals [8, 9], giving a homozygous genotype frequency at birth of 0.25% to 1 in 10,000. Full penetrance would suggest CANVAS to be a more common disease than is currently recognised. Although misdiagnosis of this recently described syndrome appears common, the true prevalence of *RFC1-*related disease is uncertain [27].

## *RFC1*-Associated Cough

### Chronic Cough in CANVAS

Chronic cough was recognised to be common in CANVAS from the earliest descriptions of the syndrome [26]. Noteworthy is the fact that the cough often predates other clinical features by many years, possibly even decades, with an age of onset of 30 years or younger [31]. One case series comprised five non-smoking subjects with CANVAS who initially experienced chronic cough up to 29 years prior to the onset of neurological symptoms. The cough was typically dry, and triggered by factors including bending down, strong odours, and eating dry foods. Typical symptoms consistent with gastro-oesophageal disease were present in only one individual, none were taking angiotensin-converting enzyme-inhibitor medication, and there was no evidence of respiratory disease other than the cough [31].

A recent larger series of 13 patients reported similar findings, but cases were genetically confirmed, being investigated following the identification of the association of *RFC1* with CANVAS [46]. In this series, in five of seven subjects tested with either barium swallow oesophageal manometry there was some evidence of oesophageal dysmotility, but empirical treatment for gastroesophageal reflux with proton pump inhibitor or H_1_-receptor blocker medication consistently failed to improve the cough. Neuromodulator medication (amitriptyline, gabapentin or pregabalin) was trialled for cough in eight patients, with only three reporting any benefit. Cough-related quality of life (QoL) as measured with the Leicester Cough Questionnaire was substantially reduced at a mean score of 11.6 points (out of a maximum score for QoL of 21) [46].

In a specialist cough clinic in London, eight patients with neurologist-diagnosed CANVAS with coexistent chronic cough, genetically confirmed with biallelic repeat expansions in *RFC1*, were compared to 53 sequential patients with well-characterised UCC or RCC [47]. In all, potential contributory common respiratory and non-respiratory pathology and factors (including asthma and gastroesophageal reflux) had been considered and either effectively excluded or addressed without successful treatment of the cough. None of the patients without CANVAS had clinical evidence of peripheral or autonomic neuropathy. In terms of patient-reported cough severity (measured with a visual analogue scale), triggers (measured with the Cough Hypersensitivity Questionnaire, CHQ), and cough reflex sensitivity (measured with inhalational capsaicin tussive tests)*,* there was no difference in the characteristics of the cough between groups. Patient-rated cough was consistently of high severity, average CHQ scores similarly high, and capsaicin concentration thresholds to induce coughing almost universally low. Daily cough frequency (measured objectively with a portable cough monitor) was significantly raised in the eight patients with CANVAS, although not as high as in the larger general specialist cough clinic group with RCC/UCC.

There was a 3-to-1 female predominance in the group without CANVAS, whereas 4 of the 8 patients with CANVAS-cough were female. Of particular note, consistent with other reports, was the much young median age of onset of 33 years for CANVAS-cough, and 55 for refractory chronic cough in the other group [47].

### *RFC1* in Refractory Chronic Cough

Guilleminault et al*.*, in a respiratory clinic in Limoges, France studied 68 patients with well-characterised RCC, none of whom had ataxic or vestibular symptoms [10]. Genotyping identified the presence of biallelic pathogenic repeat expansion *RFC1* variants in 11 (16%). A further six individuals (9%) had monoallelic variants indicating carrier status. Those with (mono- or biallelic) *RFC1* repeat expansions were younger with mean age at the onset of cough of 45 years, compared to 51 years in wild-type *RFC1* variants. Clinical characteristics across the two groups were otherwise similar. These included sex, body mass index, comorbidities, lung function, subjective descriptions and severity of the cough, and LCQ scores. Food and dust or smoke as triggers of cough seemed more frequent in those with variant *RFC1*, although chance associations in this study is possible given the testing of multiple hypotheses. At the time of the study the mean age of those with repeat expansion *RFC1* was 59 years [10].

As discussed below, these findings are remarkable for the implication that *RFC1* polymorphism is a potential significant cause of chronic cough, being present in a sizable minority in this cohort, and presumably a precursor of neurological disability in at least some. However, the authors’ suggestion that a heterozygous repeat expansion *RFC1* genotype may lead to chronic cough as well as biallelic variant *RFC1* is far from clear cut. A heterozygote (carrier) frequency of 9% is unusually high if the background heterozygote frequency is taken to be 0.7%, as quoted in the original work by Cortese et al. [8]. However, subsequent work including subjects of mixed European and non-European ancestry has estimated a background carrier frequency of *RFC1* intronic AAGGG repeat expansion as closer to 5% [9]. This latter value is not significantly different from the proportion of heterozygotes in the study of Guilleminault et al. given the small sample size. The possibility that monoallelic *RFC1* AAGGG repeat expansion may lead to disease cannot be excluded, but the only circumstance in which this has been demonstrated to date is as part of a compound heterozygous genotype in combination with an *RFC1* nonsense mutation sequence [48]. Further data are needed.

## Unifying Concepts

Chronic cough has been noted previously in association with other inherited neurological disorders, including autosomal dominant hereditary sensory neuropathy type 1 (HSN1) [49], Charcot-Marie-Tooth (CMT) disease [50], and Holmes Adie syndrome [51]. The underlying mechanism of cough in these conditions is unclear. In patients with HSN1 and cough, gastroesophageal reflux disease (GORD) was reported as a common, but not universal, feature and there was little correlation between GORD severity and cough [49]. It seems GORD does not fully explain all cough in these patients, although may be an aggravating factor.

Caution, however, is needed in ascribing cough to conditions such as HSN1, as the discovery of *RFC1* expansions has prompted further investigation and revision of many neurological diagnoses to CANVAS or *RFC1* disorders [52, 53]. Beijer et al. reported that 9 of 12 families with diagnosed HSN and cough, with unremarkable whole-exome sequencing, had biallelic *RFC1* expansions [53]. Kumar et al. reported that 2 of 4 families initially diagnosed with HSN were found to have biallelic *RFC1* expansions, but this was not evident in those with HSN and cough linked to chromosome 3p22-p24 [52]. Thus, whilst *RFC1* expansions may be the underlying genetic cause of many neurological conditions with cough, it seems probable that a number of different, hitherto undiscovered, genetic aetiologies are at play.

The identification of cough hypersensitivity as a common feature of a distinct neurological syndrome with a genetic basis gives strong support to the concept of the cough hypersensitivity syndrome in general, essentially a hypothesis supported by clinical observation [13]. It also gives weight to the idea of cough as a neuropathic disorder [5]. Furthermore, evidence for a genetic basis to refractory chronic cough in strong association with ‘hard’ clinical signs and neurophysiological features of disease gives weight to UCC/RCC as a tangible clinical entity in its own right. This should help patients with UCC/RCC give meaning to their condition and validate their concerns that they have a ‘real’ medical condition, a condition which is poorly understood by laypeople and medical professionals alike. In turn, wider evidence in support of the entity of refractory chronic cough due to underlying cough hypersensitivity will hopefully provide further justification for clinical guidelines which advocate for less unwarranted low-yield investigation for secondary causes for cough, and for fewer blind ‘empirical’ trials of treatment of conditions sometimes associated with cough, such as gastro-oesophageal reflux or sinus disease, in the absence of specific features of those conditions [3].

## Implications and Questions for Clinical Practice and Research

**Fig. 1:**
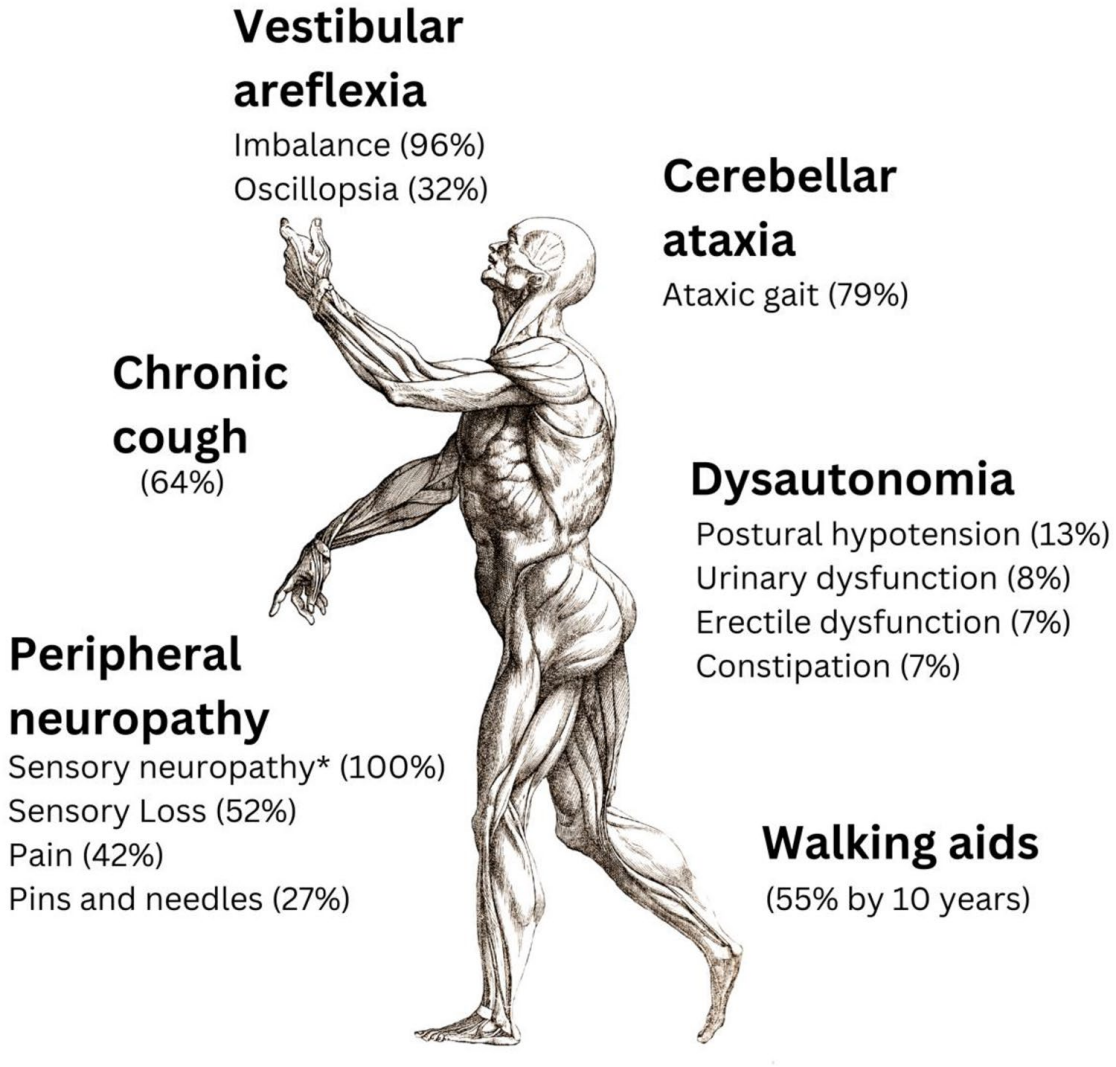
Clinical features of CANVAS. Data presented as overall prevalence. 100% patients with CANVAS experience > 1 symptom. *Based on nerve conduction studies. Adapted from Ref*.* [27]

The observation of pathogenic *RFC1* repeat expansion sequences at a significantly raised frequency in patients with refractory or unexplained cough needs independent corroboration from investigation in other settings [10]. However, regardless of the absolute prevalence of biallelic repeat expansion *RFC1* in chronic cough, clinicians need to be aware of CANVAS, particularly in the presence of certain neurological clinical features, and with a family history of unsteadiness or chronic cough in siblings (see Fig. 1). Part of routine history taking in UCC/RCC should therefore as a minimum in this regard specifically include questions about family history, sensory loss and/or paraesthesia in the extremities, and unsteadiness or falls, particularly in the dark. Earlier identification of the syndrome should prompt consideration of nerve conduction studies and referral to a neurology clinic for specialist advice and management, possibly including genetic testing (Table 1).

A question may arise as to whether *RFC1* testing has a place in the investigation of refractory chronic cough. At least for now, particularly in the absence of additional clinical features of CANVAS, it is probably too soon to advocate for this routinely whilst many questions remain. As above, the findings of Guilleminault et al. need to be replicated and expanded to understand the prevalence of *RFC1* expansions, not only in refractory chronic cough, but in cough secondary to other respiratory diseases. The implication of a single copy of repeat expansion variant requires further investigation. Having said this, if *RFC1* genotyping was more readily available, the lack of distinguishing features for *RFC1*-cough may prompt consideration of routine genetic testing in RCC/UCC, if not least to understand more about *RFC1* polymorphism in relation to cough.

More broadly, the specific mechanisms underlying cough in CANVAS, and other neurological disorders, needs to be better understood. The long latency period between cough and the development of other features in this inherited condition suggests the neuronal pathways regulating the cough reflex have a particular vulnerability to the effects of *RFC1* expansions, and potentially also to the general effects of ageing. It is not yet known if specific changes occur in the innervation of the airways, in terms of either afferent nerve density or function. The presence of autonomic features in CANVAS, and of vagal nucleus changes on autopsy [36], suggest vagal dysfunction could also be at play. Also, although the cerebral cortex seems to be largely unaffected in the syndrome, alterations in central pathways moderating the cough reflex remains a possibility.

Improved understanding of *RFC1*-related disease would have implications for chronic cough more generally, and could lead to new approaches to treatment.

## Conclusion

The identification of refractory chronic cough with features of cough reflex hypersensitivity as a common feature of CANVAS, a distinct neurological syndrome caused by biallelic AAGGG expansions in the DNA replication and repair gene *RFC1,* is an exciting development with wide implications for clinical management and research.

## Data Availability

Data sharing is not applicable to this article as no new data were created or analysed in this review.
